# Polyethylene Glycol/Pullulan-Based Carrier for Silymarin Delivery and Its Potential in Biomedical Applications

**DOI:** 10.3390/ijms25189972

**Published:** 2024-09-16

**Authors:** Julia Iwaniec, Karina Niziołek, Patryk Polanowski, Dagmara Słota, Edyta Kosińska, Julia Sadlik, Krzysztof Miernik, Josef Jampilek, Agnieszka Sobczak-Kupiec

**Affiliations:** 1 Cracow University of Technology, Faculty of Materials Engineering and Physics, Department of Materials Science, 37 Jana Pawła II Av., 31-864 Krakow, Poland; 2 Cracow University of Technology, CUT Doctoral School, Faculty of Materials Engineering and Physics, Department of Materials Science, 37 Jana Pawła II Av., 31-864 Krakow, Poland; 3 Department of Analytical Chemistry, Faculty of Natural Sciences, Comenius University, Ilkovicova 6, 842 15 Bratislava, Slovakia; 4 Department of Chemical Biology, Faculty of Science, Palacky University Olomouc, Slechtitelu 27, 783 71 Olomouc, Czech Republic

**Keywords:** polymers, flavonoids, silymarin, pullulan, polyethylene glycol

## Abstract

Restoring the structures and functions of tissues along with organs in human bodies is a topic gathering attention nowadays. These issues are widely discussed in the context of regenerative medicine. Excipients/delivery systems play a key role in this topic, guaranteeing a positive impact on the effectiveness of the drugs or therapeutic substances supplied. Advances in materials engineering, particularly in the development of hydrogel biomaterials, have influenced the idea of creating an innovative material that could serve as a carrier for active substances while ensuring biocompatibility and meeting all the stringent requirements imposed on medical materials. This work presents the preparation of a natural polymeric material based on pullulan modified with silymarin, which belongs to the group of flavonoids and derives from a plant called *Silybum marianum*. Under UV light, matrices with a previously prepared composition were crosslinked. Before proceeding to the next stage of the research, the purity of the composition of the matrices was checked using Fourier-transform infrared (FT-IR) spectroscopy. Incubation tests lasting 19 days were carried out using incubation fluids such as simulated body fluid (SBF), Ringer’s solution, and artificial saliva. Changes in pH, electrolytic conductivity, and weight were observed and then used to determine the sorption capacity. During incubation, SBF proved to be the most stable fluid, with a pH level of 7.6–7.8. Sorption tests showed a high sorption capacity of samples incubated in both Ringer’s solution and artificial saliva (approximately 350%) and SBF (approximately 300%). After incubation, the surface morphology was analyzed using an optical microscope for samples demonstrating the greatest changes over time. The active substance, silymarin, was released using a water bath, and then the antioxidant capacity was determined using the Folin–Ciocâlteu test. The tests carried out proved that the material produced is active and harmless, which was shown by the incubation analysis. The continuous release of the active ingredient increases the biological value of the biomaterial. The material requires further research, including a more detailed assessment of its balance; however, it demonstrates promising potential for further experiments.

## 1. Introduction

Over the last two decades, there has been considerable development in the field of controlled drug delivery systems, especially those based on natural polymers. Drug delivery is defined as an approach for introducing a therapeutic agent into the body. It improves the safety, efficacy, duration, and site specificity of drug release [1,2].

**Figure 1:**
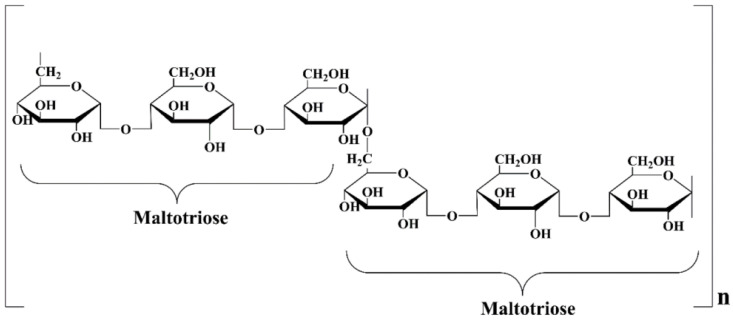
Structure of pullulan [11].

Polymers play a significant role in this regard, as they help in the transportation of the drug and act as the base material for hydrogel formation [3,4]. Hydrogels, formed from polymers, are widely used for biomedical purposes. Due to their hydrophilic structure, hydrogels are able to retain large amounts of water. They have the ability to expand to 10 to 1000 times their dry weight [5,6]. They are also characterized by high biocompatibility, non-toxicity, and porous structures. All these properties make these materials increasingly used in tissue engineering, dressings, and as drug delivery systems [7]. They are also biodegradable and biocompatible, making them suitable candidates for novel formulations [8,9]. Pullulan, a natural exopolymer, has emerged as a leader in hydrogel technology [10]. Moreover, its properties such as high biocompatibility, non-carcinogenicity, and non-toxicity emphasize its uniqueness [11]. It is commercially produced by the yeast-like fungus *Aureobasidium pullulans*, although it can be also produced by several other strains, such as *A. melanogenum, A. mousonni, Cytaria harioti, Cytaria darwinii, Termella mesenterica, Cryphonectria parasitical, Teloschistes falvicans, Rhodotorula bacarum, Cryphonectria parasitica, Eurotium cheyalieri*, *E. cheyalieri, Aspergillus japonicus*, and *Rhodosporidium paludigenum* [12]. It is highly biocompatible, non-toxic, and notably devoid of any mutagenicity or immunogenicity. Pullulan’s unique properties make it a potential candidate for biomedical applications, particularly in drug delivery, gene delivery, tissue engineering, and regenerative medicine [10,13]. Due to its properties such as low toxicity, biodegradability, adequate adhesion, and oxidative properties, pullulan is used for controlled and targeted drug release. The presence of nine chemically active hydroxyl groups in the structure of the pullulan molecule allows for its modification and thus the introduction of active substances, e.g., silymarin [14]. Its unique structure consists of repeating maltotriose units linked by α-(1→4) and α-(1→6)-glycosidic bonds [15,16] (Figure 1).

Another important polymer widely used in the biomedical field is polyethylene glycol (PEG). This hydrophilic polymer has properties such as non-toxicity, non-immunogenicity, and biocompatibility that make it applicable to tissue engineering as well as drug delivery systems. Its popularity as a drug carrier is related to its active hydroxyl end, which allows it to bind to the active drug molecule. Due to their many advantages, PEG-based hydrogels have a wide range of applications in the biomedical and pharmaceutical fields [17].

Many compounds can act as drug carriers, but flavonoids are currently attracting particular attention. These are polyphenolic compounds that can be divided into six main groups: isoflavonoids, flavanones, flavanols, flavonols, flavones, and anthocyanidins [18,19]. Flavonoids were discovered in 1930 by the Hungarian scientist Albert Szent-Györgyi, who isolated a new chemical substance from oranges during an experiment [20]. To date, over 8000 different flavonoids have been described [21]. They comprise one of the most numerous and widely distributed groups of secondary metabolites [22,23]. They occur in food sources such as vegetables, fruits, wine, and even tea [24]. Their main effects are related to health benefits, including anticancer and antiviral properties. Recently, interest in these compounds has grown significantly, particularly due to their antiviral activity, which has become increasingly important given the rising incidence of viral infections, especially influenza, in recent times [25,26]. Flavonoids also have anti-inflammatory, vasodilatory, antithrombotic, cardioprotective, antidiabetic, neuroprotective, and anti-obesity effects [27]. Interestingly, they also have a positive effect on stopping the aging process, improving physical function [28,29].

In terms of flavonoid carriers, silymarin is an interesting solution. It is a substance extracted from the plant *Silybum marianum* (L.) Gaertn., popularly known as milk thistle [30,31]. It can be found in Kashmir, North America, Canada, and Mexico. It usually has large leaves and reddish-purple flowers, which are prickly. The medicinal part of milk thistle is the seeds or fruits. It has been used for over two thousand years to treat many ailments, such as liver diseases [32,33].

Its main pharmacological advantages include hepatoprotective, anti-inflammatory, antioxidant, anticancer, and cardioprotective effects [34]. As a polyphenolic complex, it can penetrate the body through the blood–brain barrier (BBB) to realize its potential. Even in high doses, it does not cause any undesirable side effects in either humans or animals, and its safety is well documented [35,36]. The properties of silymarin have been tested in various in vitro systems based on different cells, as described extensively by Peter F. Surai et al. Its bone-protective and anti-inflammatory properties have been proven [37].

The aim of this work is to develop a hydrogel material based on pullulan, a natural polymer, that will be able to carry the active substance silymarin. The hydrogel material produced is particularly suitable for use as a dressing material. The polymer used is an excellent alternative to dressings thanks to its structure, additionally providing wound soothing, antibacterial properties, and non-immunogenicity. Moreover, the enrichment of the dressing with flavonoids, e.g., silymarin, is desirable for wound treatment. Silymarin can improve the action of the dressing due to its properties. It is a powerful antioxidant that is responsible for scavenging free radicals and has anti-inflammatory properties, which can facilitate the wound healing process in the case of wound infection. The main application of the manufactured biomaterial is dressings, but it should be noted that the combination of pullulan and silymarin is excellent for application as a drug delivery system. This is possible thanks to the structure of pullulan, which allows it to be modified and thus charged with drugs or active substances [38,39]. The polymer phase was additionally enriched with polyethylene glycol, which is a linear polymer with chemically active hydroxyl groups at both ends, which enables easy conjugation with functional groups [40,41]. It increases the half-life and effectiveness of therapeutic molecules [42]. Physicochemical analysis and incubation studies lasting 19 days were performed on the prepared materials. The sorption capacity of the prepared samples was determined. The pH and electrolytic conductivity were measured, and purity was checked by FT-IR spectroscopy. The release profile of the active ingredient, silymarin, was examined. The surface morphology of the finished matrices was examined by optical microscopy, which is of particular importance in the context of regenerative medicine.

## 2. Results

### 2.1. In Vitro Incubation

#### 2.1.1. Determination of Sorption Capacity

After 15 min, 1 h, 2 h, 24 h, 5 days, 7 days, 15 days, and 19 days, the sample weight was measured by draining off excess fluid using filter paper. The measurement was repeated analogously for each matrix. The sample labels in the legends of the graphs are analogous to the labels of the compositions of the prepared materials (Table 1).

**Figure 2:**
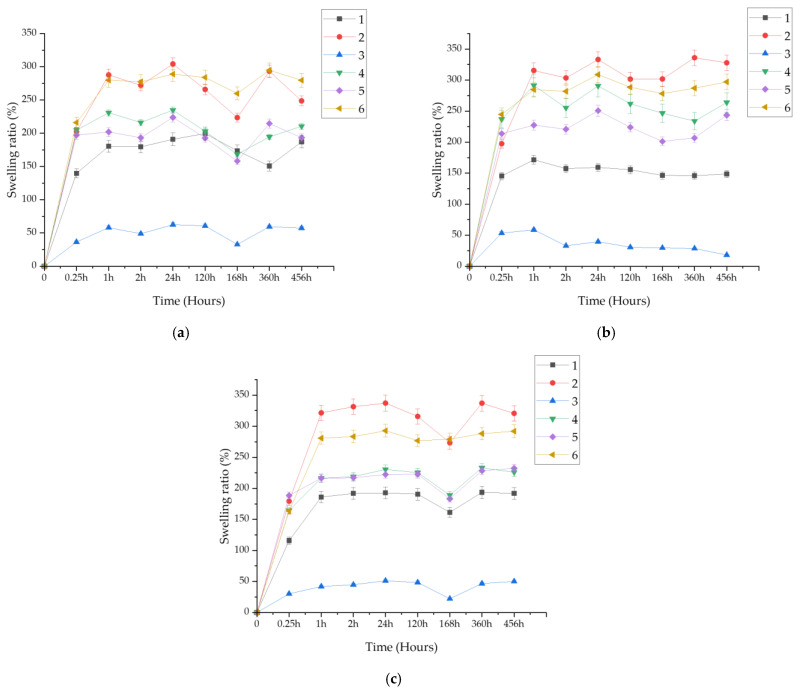
Sorption capacity for samples in: (**a**) SBF; (**b**) artificial saliva; (**c**) Ringer’s solution.

Figure 2 presents the time dependence of the swelling coefficient of the formed biomaterials in fluids simulating the body’s internal environment, i.e., SBF, artificial saliva, and Ringer’s fluid. Sample 2, composed of 10 mL of pullulan, 1 mL of polyethylene glycol with a molecular weight of 400, 2 mL of a crosslinking agent with a molecular weight of 700, and 50 μL of a photoinitiator, demonstrated the highest sorption capacities across all the fluids. This sample contained the highest amount of pullulan, which positively influenced its sorption abilities. In contrast, Sample 3 exhibited the lowest sorption capacities among all the samples. Its results significantly differed from the others. This material comprised 11 mL of polyethylene glycol with a molecular weight of 400, 2 mL of a crosslinking agent with a molecular weight of 700, and 50 μL of a photoinitiator. It lacked pullulan and polyethylene glycol with a molecular weight of 6000, leading to a considerable reduction in sorption capacities. Notably, samples submerged in Ringer’s solution and artificial saliva (approximately 350%) demonstrated notably higher sorption capacity than those in SBF (approximately 300%). In SBF, due to the high presence of ions reacting with the material and forming crosslinks with polymer chains, the biomaterials were likely more crosslinked than in other fluids, resulting in decreased sorption capacities.

#### 2.1.2. pH Metric Analysis

**Figure 3:**
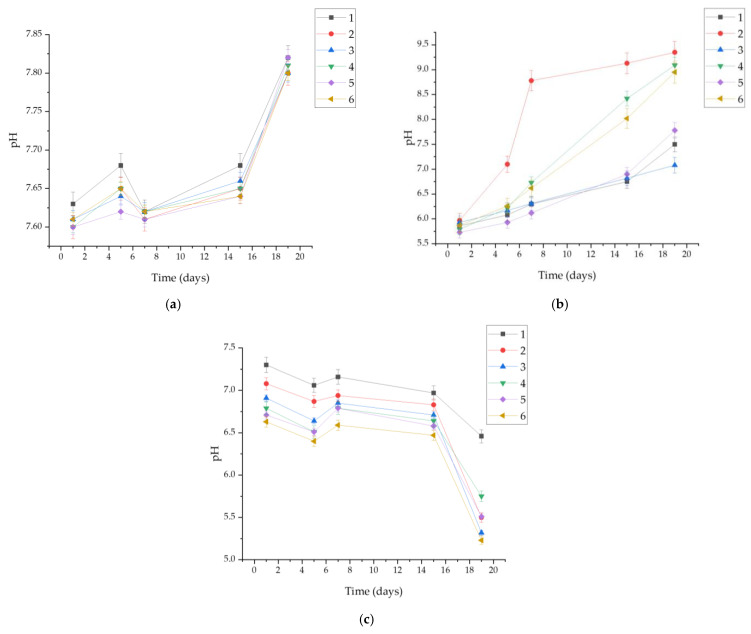
Measured pH values for samples in: (**a**) SBF; (**b**) artificial saliva; (**c**) Ringer’s solution.

A pH level test was performed to determine the effect of the biomaterials on the physiological environment. Changes in pH levels can determine the release of ions into the incubation medium. Three fluids were used to provide different environments for the materials. SBF fluid is a strong buffer. Its ability to maintain the environment at a constant pH of around 7.4 is similar to the function of blood in the human body. Artificial saliva of a completely different nature was also used; its pH is lower, at around 5.5 pH. It is a more aggressive environment than the aforementioned SBF, as confirmed by the experiment. The pH level changed significantly, rising from 5.5 to 9, confirming a strong ion exchange between the material and the medium. In this fluid, the tested composite was the least stable. The third medium used was Ringer’s liquid, with a pH of about 6.5. These fluids, which are different in nature, provided the widest possible spectrum of measurements and allowed the behavior of the composite to be studied under different conditions. The analysis of pH value changes is presented in Figure 3 for the tested materials in fluids simulating the body’s internal environment. Monitoring changes over time plays a crucial role, especially when the materials are considered for medical applications. The most stable pH was observed in SBF. Throughout the observation period, it remained within the range of 7.6–7.8, which, compared to Ringer’s solution (5.1–7.6) and artificial saliva (5.6–9.6), represents a stabilized result. This is attributed to the buffering properties of SBF. The most significant pH changes and fluctuations over time were observed in the artificial saliva. Values increased significantly with time. The highest pH value of 9.6 was recorded for Sample 2. After 5 days, matrix degradation in the fluid was also noticeable. This was likely associated with the presence of sulfate ions. In Ringer’s solution, the pH remained stable for each sample over 15 days. After this period, a sudden decrease was observed, reaching a value of 5.1 for Sample 6.

#### 2.1.3. Conductivity Analysis

**Figure 4:**
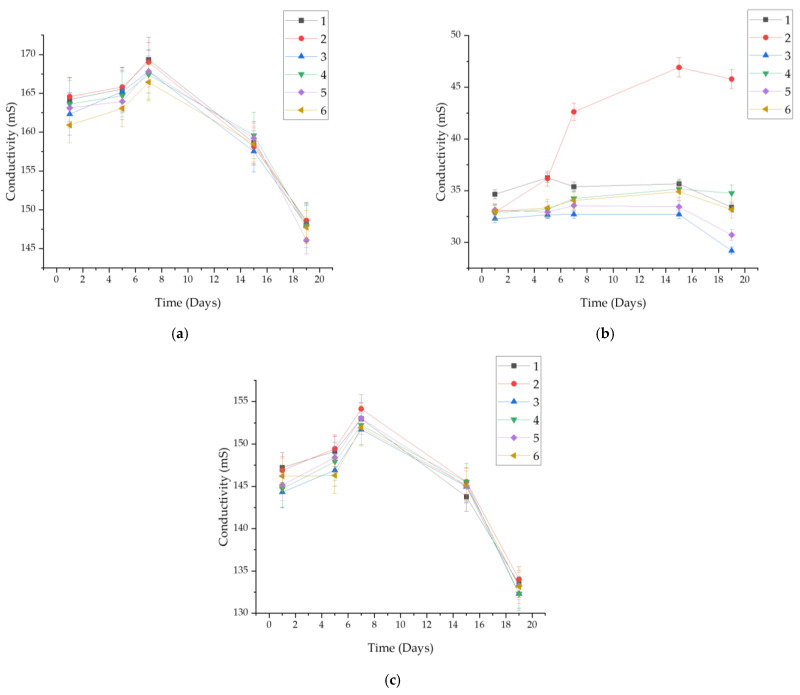
Measured conductivity values for samples in: (**a**) SBF; (**b**) artificial saliva; (**c**) Ringer’s solution.

Like the observation of pH changes, electrolytic conductivity was also examined. Figure 4 shows a graph of changes in electrical conductivity over time in the fluids.

The highest electrical conductivity was exhibited by samples immersed in SBF, ranging from approximately 145 to 170 mS. This is due to its rich ionic composition. Conductivity increased until day 5 of observation, after which a decrease was noticeable for each sample. In Ringer’s solution, conductivity ranged within 130–155 mS. A decline was observed after 7 days from the start of the observation. The samples behaved similarly over time. The lowest electrical conductivity was seen in artificial saliva, ranging within 25–50 mS, which is significantly lower than that in the other fluids. Additionally, the conductivity graph for Sample 2 presents markedly higher values compared to those for the other samples. This was caused by the gradual degradation of the biomaterials.

### 2.2. Fourier-Transform Infrared Spectroscopy Analysis

#### Spectrum of Biomaterials before Incubation

**Figure 5:**
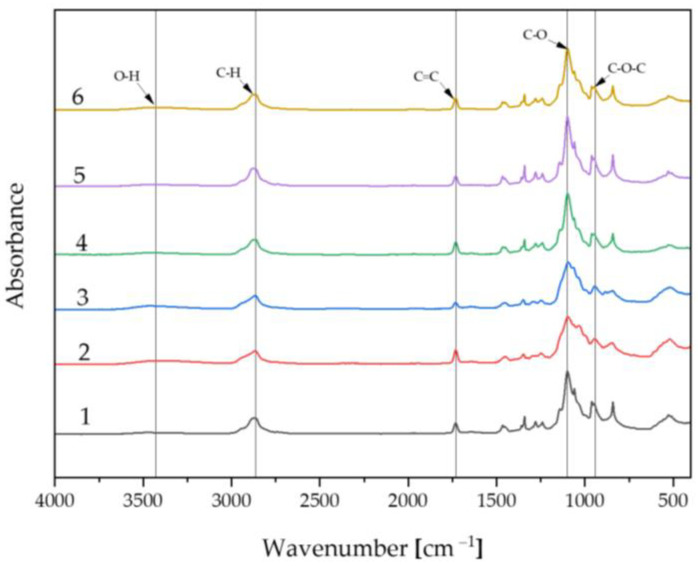
FT-IR spectra of biomaterials before incubation.

The FT-IR spectrum is presented in Figure 5, which demonstrates the relationship between absorbance and wavelength in the 4000–400 cm⁻¹ range. The spectra of the individual samples are similar and overlap. The compositions of the prepared materials are pure and consistent. The analysis clearly indicates that the highest characteristic peak common to each sample is around 1098 cm⁻¹ and is associated with the C-O functional unit, representing the pullulan polymer. Bands in the range of 1731 cm⁻¹ are linked to the C=C moiety. In the wavelength range of 2865–2875 cm⁻¹, the C-H functional group can be observed. Additionally, emerging peaks in the range of 3000–3500 cm⁻¹ represent O-H, and bands at 943–962 cm⁻¹ correspond to the C-O-C moiety [43,44].

### 2.3. Determination of Release Kinetics of Silymarin

#### Spectrophotometer Analysis

**Figure 6:**
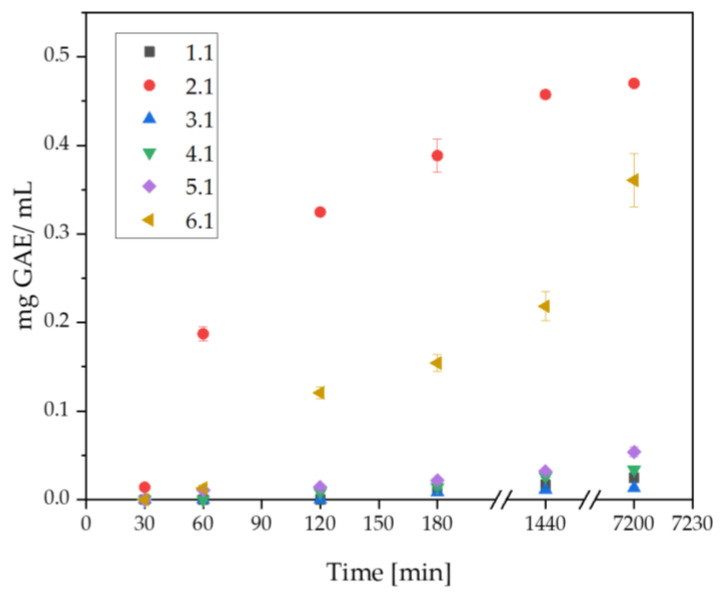
Analysis of changes in the amount of silymarin released over time.

The release profile of the active ingredient, which was silymarin, is presented in Figure 6. The silymarin contained in the produced materials was active. The highest concentration of released silymarin, observed after 5 days, was found in Sample 2.1 (0.470 mg/mL), which was a material consisting of 10 mL pullulan, 5 mg silymarin dissolved in 1 mL polyethylene glycol with a molecular weight of 400, 2 mL crosslinking agent with a molecular weight of 700, and 50 μL photoinitiator. A high concentration was also observed in Sample 6.1 (0.361 mg/mL), composed of 2.5 mL polyethylene glycol with a molecular weight of 6000, 7.5 mL pullulan, 5 mg silymarin dissolved in 1 mL polyethylene glycol with a molecular weight of 400, 2 mL crosslinking agent with a molecular weight of 700, and 50 μL photoinitiator. Both materials shared the highest pullulan content, which positively influenced the release rate of silymarin. The active substance was detectable as early as 1 h. In other samples, the release started after 3 h. The lowest amount of silymarin detected after 5 days was in Sample 3.1, a biomaterial consisting of 5 mg silymarin dissolved in 11 mL polyethylene glycol with a molecular weight of 400, 2 mL crosslinking agent with a molecular weight of 700, and 50 μL photoinitiator. The absence of pullulan and polyethylene glycol with a molecular weight of 6000 negatively affected the final result. The obtained biomaterials can serve as carriers for active substances in regenerative medicine applications.

### 2.4. Morphology Analysis

#### 2.4.1. Material Morphology before Incubation

**Figure 7:**
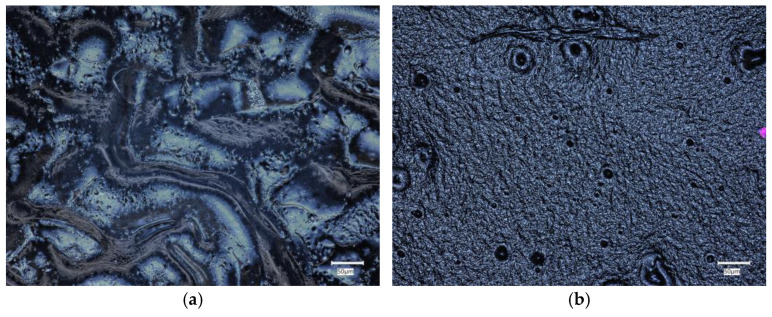
Optical microscope images of (**a**) Sample 2; (**b**) Sample 6.

Surface morphology analysis was conducted for samples exhibiting the highest sorption capacities and the largest amount of released silymarin, namely, Samples 2 and 6. Structural images were captured using an optical microscope both before and after incubation in SBF, Ringer’s solution, and artificial saliva. Figure 7 presents images of the surface morphology before incubation. Before the incubation process, the surface of Samples 2 and 6 exhibited a relatively smooth appearance, characterized by minimal irregularities or surface features.

#### 2.4.2. Material Morphology after Incubation

Samples immersed in SBF exhibited the most stable structure. This was attributed to the specificity of this fluid, which mimics the organic ion concentration in human blood plasma. In Ringer’s solution, numerous stratifications were visible, which led to a reduction in sample volume during the assessment of sorption capacities. The samples also underwent slow degradation. In artificial saliva, degradation was also noticeable, progressing with incubation time, stemming from the nature of the fluid. However, it occurred much faster than in Ringer’s solution.

**Figure 8:**
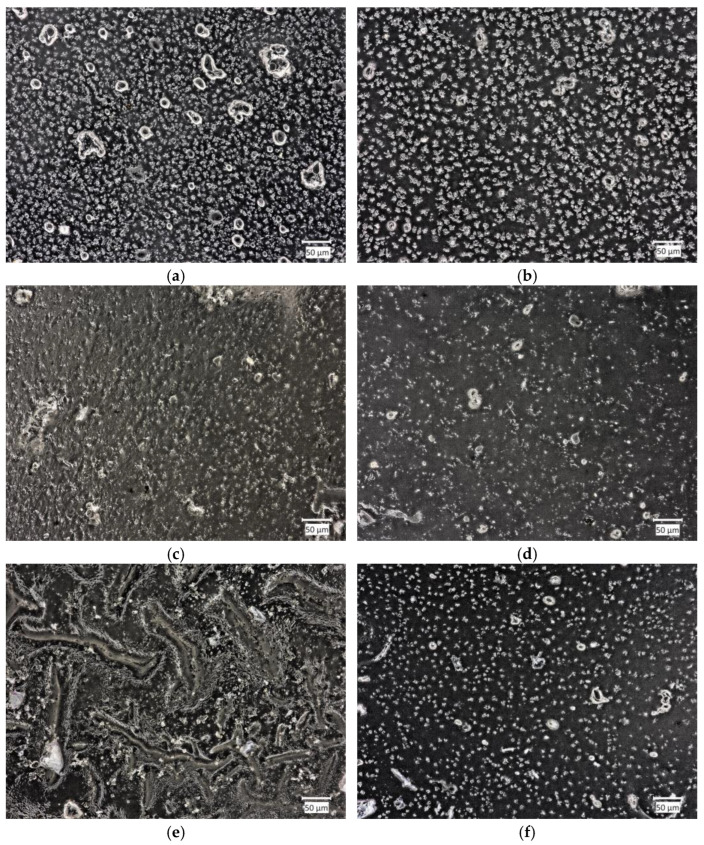
Optical microscope images of: (**a**) Sample 2 in Ringer’s solution; (**b**) Sample 6 in Ringer’s solution; (**c**) Sample 2 in SBF; (**d**) Sample 6 in SBF; (**e**) Sample 2 in artificial saliva; (**f**) Sample 6 in artificial saliva.

Figure 8 shows images of surface morphology after incubation. The surfaces of Samples 2 and 6 were examined after the incubation process in artificial saliva, Ringer’s solution, and SBF.

## 3. Discussion

Regenerative medicine aims to unravel the intricate processes of natural regeneration observed in various organisms. By delving into the mechanisms that govern regeneration in biological systems, regenerative medicine seeks to harness this knowledge to develop innovative therapeutic strategies that can promote tissue repair and restoration in human patients [45]. The physicochemical and incubation studies conducted allowed for the evaluation of the developed biomaterials as well as their validation for future biomedical applications. The proposed method for obtaining hydrogels enables researchers to obtain continuous, fully crosslinked materials. FT-IR analysis confirmed the purity of the starting components, demonstrating that they were phase pure and had the appropriate functional groups. This made it possible to take further steps toward physicochemical evaluation. Incubation studies were conducted, during which pH and ionic conductivity were measured. In the case of pH, the greatest changes were observed in artificial saliva; considering the nature of the composition of this fluid, with its slightly acidic initial pH, this may indicate partial degradation of the polymer matrix. The most stable pH was observed in SBF (7.6–7.8), which may be due to the buffering nature of this fluid. In the case of ionic conductivity, the values for all fluids increased comparably. In the case of artificial saliva, this is directly related to material degradation, while in the case of SBF and Ringer’s fluid, it indicates interactions occurring between the ions in the fluids and the material. This is a desirable phenomenon, as the absence of interactions would indicate that the material is inert, which is not positive for biomaterials, especially those with the desired bioactivity. Interactions were also confirmed by microscopic observations of the surface morphology, where changes were observed. The entire surface was covered with blooms, new crystals that were formed by the aggregation of individual ions. Additional EDS analysis is necessary to assess the nature of these crystals; however, given the composition of the fluids, they are likely to be sodium or potassium chlorides, or calcium phosphates in the case of SBF. The sorption capacity of the materials was also evaluated during the incubation tests. For all samples, an increase of up to more than 300% was observed. This is important, as even the smallest sorption capacities confirm the potential use of the material as a drug carrier. Determining the sorption capacity of the material plays a significant role in assessing fluid absorption and, simultaneously, material swelling. The specified chemical composition of the reagents not only affects the structure but also the swelling process [46]. A dependence of the behavior on the composition was observed. Samples 2 and 6 swelled the most, and the highest amount of pullulan was found in them. It is interesting to note that the same samples caused the greatest increase in pH values when incubated in artificial saliva. Sample 3, without pullulan, demonstrated the lowest sorption capacity. This therefore indicates that the chosen polysaccharide was the main determinant of the swelling capacity and overall behavior of the materials in an aqueous environment. The swelling parameter was closely related to the ability to release the drug. Samples 2 and 6 released the most silymarin over time. The obtained hydrogel biomaterials based on pullulan can serve as carriers for active substances, including various drugs or flavonoids, in regenerative medicine applications. The use of silymarin in pullulan-based systems with immunomodulatory properties has been studied previously by Santhos Kumar et al. 2012. Pullulan and the active substance were combined in a completely different way. In this study, silymarin was encapsulated in pullulan acetate, and then the release of the drug was checked. It was found that after 24 h, the release of silymarin was at such a high level that it was capable of stopping the growth of liver cancer cells. These results indicate an excellent application of pullulan for drug release systems, including silymarin, which was also used in our study [47]. The applications of regenerative medicine technology may offer modern therapies for patients with injuries, end-stage organ failure, or other clinical problems [48]. Complex of modified pullulan, conjugated with targeted active substances, exhibits high bioactivity and acts as a good carrier for controlled drug or gene release [49].

## 4. Materials and Methods

### 4.1. Materials

Polyethylene glycol (PEG, Mn = 6000, concentration 20%, CAS 25322-68-3) from Acros Organic (Geel, Belgium) was used for the chemical synthesis of the biomaterials; poly(ethylene glycol) diacrylate (PEGDA, Mn = 700, CAS 26570-48-9) from Sigma Aldrich (Darmstadt, Germany) was used as a crosslinking agent; and 2-hydroxy-2-methylopropiophenone (CAS 7473-98-5) from Sigma Aldrich (Darmstadt, Germany) was used as a photoinitiator. Silymarin (CAS 65666-07-1) from Angene Chemical (Nanjing, China) with a purity of 80% was dissolved in polyethylene glycol (PEG, Mn = 400, CAS 25322-68-3) from TCI Chemicals (Brussels, Belgium). Pullulan (CAS 9057-02-7) with a molecular weight of 532.4902 g/m from Angene Chemical (Nanjing, China) with a concentration of 2% was used as a polymer base.

The following reagents were used to create incubation fluids, including SBF, Ringer’s fluid, and artificial saliva: sodium chloride (CAS 7647-14-5), potassium chloride (CAS 7447-40-7), and calcium chloride (CAS 10043-52-4) from Eurochem BGD (Tarnów, Poland); sodium hydrogen phosphate monohydrate (CAS 7558-79-4), sodium sulfide nonahydrate (CAS 1313-84-4), potassium hydrogen phosphate trihydrate (CAS 16788-57-1), magnesium chloride hexahydrate (CAS 7791-18-6), hydrochloric acid (CAS 7647-01-0), sodium sulfate (CAS 7757-82-6), and tris(hydroxymethyl)aminomethane (CAS 77-86-1) from Chempur (Piekary Śląskie, Poland); and urea (CAS 57-13-6) from Stanlab (Lublin, Poland).

To perform the Folin–Ciocâlteu test, a saturated solution of sodium carbonate, Folin–Ciocâlteu’s reagent, and ethanol 96% (CAS 64-17-5) from Chempur (Piekary Śląskie, Poland) and a 5 mg/mL solution of gallic acid (CAS 149-91-7) from POCH (Gliwice, Poland) were used.

### 4.2. Methods

#### 4.2.1. Preparation of Biomaterials

Pullulan (2% (*w*/*w*)) and PEG (Mn = 6000, 20% (*w*/*w*)) were prepared by mixing the polymer powder with distilled water. Then, 2 mL of PEGDA was added as a crosslinking agent. It was used to create a more rigid structure of the biomaterial and made it possible to obtain the intended shape. Pullulan was mixed with PEG 6000 in order to make the sample more flexible. Moreover, the addition of PEG to pullulan increases its solubility in organic solvents. Silymarin was dissolved in PEG (Mn = 400) with the help of ultrasound and added to the previously prepared mixture. Then, 50 µL of photoinitiator 2-hydroxy-2-methylpropinophenone was added using a pipette. The exact compositions of the matrices are presented in Table 1. The resulting mixtures were thoroughly mixed using a magnetic stirrer and then placed on a Petri dish. Chemical synthesis was carried out using a UV lamp (Medilux UV 436 HF, 220 V, 60 Hz), (Medilux, Korntal-Münchingen, Germany). The process lasted 4 min. After this time, the samples were set aside to dry and prepare for further testing.

**Figure 9:**
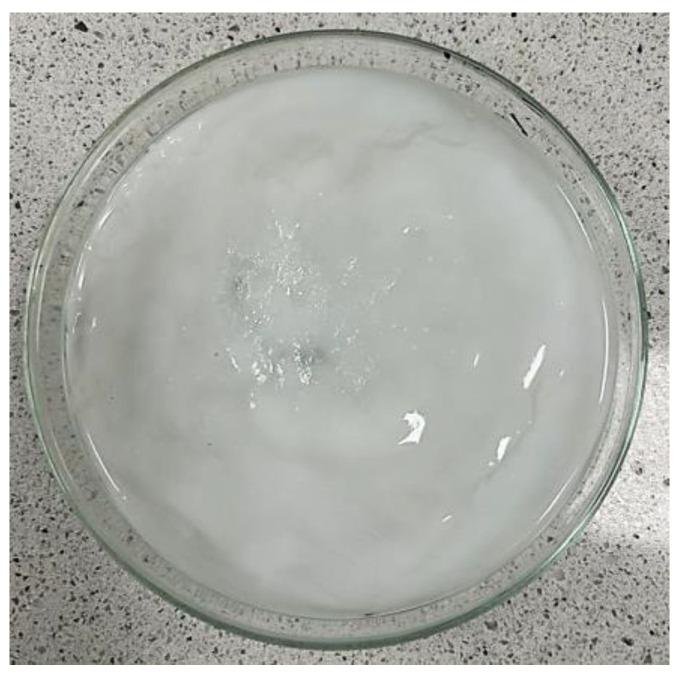
Sample 4 appearance.

A total of twelve different matrix compositions were developed. The amounts of individual reagents were manipulated in order to compare the results obtained. Sample 2 and its counterpart 2.1, containing silymarin, were those containing the highest amount of pullulan. Figure 9 presents an example of a pullulan-based biomaterial as a carrier for silymarin.

#### 4.2.2. In Vitro Incubation

The obtained materials were placed in incubating fluids for a period of 19 days. For this purpose, SBF, Ringer’s solution, and artificial saliva were utilized. The individual compositions of these solutions are presented in Table 2, Table 3 and Table 4. The samples were weighed, placed in sterile containers, and then filled with 80 mL of the chosen liquid. The matrices were subsequently placed in an incubator at temperature 36.6 °C (POL-EKO, Wodzisław Śląski, Poland). The pH level and electrolytic conductivity were monitored using the multifunctional laboratory instrument Elmetron CX-701 (Elmetron, Zabrze, Poland). The synthesized biomaterials were placed in three physiological fluids, that is, SBF, Ringer’s fluid, and artificial saliva. They were placed in an incubator for 19 days, and the pH levels of the fluids in which the biomaterials were placed were measured on days 1, 5, 7, 15, and 19 of incubation. The electrode and conductivity sensor were sequentially inserted into each container with the sample submerged in the respective fluid. The device then displayed the pH value and conductivity measurement.

The findings were crucial in determining the sorption capacity of the material, allowing for the assessment of its fluid absorption ability, i.e., the swelling of the matrix. After 15 min, 1 h, 2 h, 24 h, 5 days, 7 days, 15 days, and 19 days, the sample weight was measured by draining off excess fluid using filter paper. The measurement was repeated analogously for each matrix. The results were substituted into the following equation, and then a graph depicting the swelling capacity over time in the respective incubating fluid was created:

(1) $$R_{s}=\frac{W_{t}-W_{0}}{W_{t}}\cdot 100\%$$

where *W_t_* is the weight of the swollen material sample and *W*_0_ is the initial sample weight. Furthermore, the incubation studies confirmed the interactions between the sample and the incubation medium, enabling the evaluation of their bioactivity. The pH changes over time offered a comprehensive understanding of the material’s safety and stability.

#### 4.2.3. Fourier-Transform Infrared Spectroscopy Analysis

Before incubation, the biomaterials were also subjected to FT-IR spectroscopy. The measurements were performed using a Thermo Scientific Nicolet iS5 FT-IR spectrometer (Thermo Scientific, Loughborough, UK) equipped with an iD7 ATR accessory. The spectra were recorded in the wavenumber range of 4000–400 cm^−1^ at room temperature. The purpose of the measurements was to determine the amount of light absorbed by the samples at specific wavelengths. The obtained spectra allowed for the determination of the structure, purity, and composition of specific molecular mixtures, as well as the identification of functional groups in the prepared polymer matrices.

#### 4.2.4. Determination of Release Kinetics of Silymarin

To determine the amount of silymarin released by the prepared matrices, a water bath for release testing, Electrolab EDT-08lx (Electrolab, Mumbai, India), was utilized. The release study was conducted from the whole samples, the composition of which is presented in Table 1. The materials were placed separately on six stations and then immersed in 100 mL of ultra-pure water. The test was conducted at a temperature of 36.6 °C with a stirring speed of 50 RPM. After 30 min, 1 h, 2 h, 3 h, 24 h, and 5 days, 1 mL of the liquid was withdrawn into Eppendorf tubes. These samples were then be used for antioxidant determination using the Folin–Ciocâlteu method. This involves the transfer of electrons between the Folin–Ciocâlteu reagent, an alkaline solution that produces a blue color, and phenolic compounds (present in silymarin). The color changes proportionally to their concentration. The analysis involves measuring the absorbance of the solution using a UV-Vis spectrophotometer at 765 nm. The total phenolic content was expressed as gallic acid equivalents (mg GAE, g⁻¹). In the Thermo Scientific Genesys 180 UV-Vis spectrophotometer (Thermo Scientific, Loughborough, UK), a reference cuvette containing distilled water was placed. Samples were then analyzed sequentially. For each material, three repetitions were performed. The average concentration and standard deviation were calculated.

#### 4.2.5. Morphology Analysis

Surface morphology analysis was conducted for samples exhibiting the best sorption capacities and the highest amount of released silymarin, namely, Samples 2 and 6. The structure of the samples was recorded using the Keyence VHX-7000 optical microscope (Keyence, Osaka, Japan). By utilizing visible light as the illumination source, the optical microscope is a straightforward, cost-effective, and commonly employed tool in polymer research [50].

## 5. Conclusions

The selected composition and methodology allow for the production of hydrogel polymer materials based on pullulan, acting as carriers for flavonoids such as silymarin. The optimal selection of a crosslinking agent (2 mL) and photoinitiator (50 μL) influences the polymer matrix’s ability to crosslink pullulan and limits its brittleness. The optimal amount of silymarin added to the matrices, enabling crosslinking, was 5 mg. As a result of pH metric studies, it was found that the most stable incubation fluid was SBF, maintaining a pH level of 7.6–7.8 throughout the observation period, compared to Ringer’s solution (5.1–7.6) and artificial saliva (5.6–9.6), indicating stabilized results. Incubation studies demonstrate that the developed material is non-harmful, as evidenced by pH observation results. The compositions of the matrices are pure and consistent, as confirmed by peaks obtained in the FT-IR analysis. Pullulan positively influences sorption capabilities and the release rate of silymarin. Silymarin remains active in the produced matrices. The gradual, continuous release of the active ingredient enhances the biomaterial’s biological value. Surface analysis by optical microscopy did not reveal any changes or defects in the surface structure of the materials, confirming the correctness of the chosen ingredients and synthesis method. The research proposed in this article has shown the great potential of the material in biomedical applications.

## Figures and Tables

**Table 1:** Composition of the obtained biomaterials.

Sample	20% (*w*/*w*) PEG Solution [mL]	20% (*w*/*w*) Pullulan Solution [mL]	Silymarin [mg]	PEG 400 [mL]	PEGDA 700 [mL]	Photoinitiator [μL]
1.	10	-	-	1	2	50
2.	-	10		1		
3.	-	-		11		
4.	5	5		1		
5.	7.5	2.5		1		
6.	2.5	7.5		1		
1.1	10	-	5 mg	1		
2.1	-	10		1		
3.1	-	-		11		
4.1	5	5		1		
5.1	7.5	2.5		1		
6.1	2.5	7.5		1		

**Table 2:** Composition of Ringer’s fluid.

Ringer’s Fluid	
Component	Amount [g/L]
NaCl	8.600
KCl	0.300
CaCl_2_·H_2_O	0.480

**Table 3:** Composition of SBF.

SBF	
Component	Amount [g/L]
NaCl	8.035
NaHCO_3_	0.355
KCl	0.225
K_2_HPO_4_·3H_2_O	0.231
MgCl_2_·6H_2_O	0.311
HCl 1M	39 mL
CaCl_2_	0.292
Na_2_SO_4_	0.072
Tris	6.118

**Table 4:** Composition of artificial saliva.

Artificial Saliva	
Component	Amount [g/L]
NaCl	0.400
KCl	0.400
CaCl_2_·2H_2_O	0.795
Na_2_HPO_4_·H_2_O	0.780
Na_2_S·9H_2_O	0.005
CH_4_N_2_O	1.000

## Data Availability

Data that support the findings of this study are contained within the article.

## Abbreviations

Abbreviations	Description
PEG	Polyethylene glycol
PEGDA	Poly(ethylene glycol) diacrylate
SBF	Simulated body fluid
FT-IR	Fourier-transform infrared spectroscopy
